# Genomic amplification of 9p24.1 targeting *JAK2*, *PD-L1*, and *PD-L2* is enriched in high-risk triple negative breast cancer

**DOI:** 10.18632/oncotarget.4494

**Published:** 2015-07-03

**Authors:** Michael T. Barrett, Karen S. Anderson, Elizabeth Lenkiewicz, Mariacarla Andreozzi, Heather E. Cunliffe, Christine L. Klassen, Amylou C. Dueck, Ann E. McCullough, Srikanth K. Reddy, Ramesh K. Ramanathan, Donald W. Northfelt, Barbara A. Pockaj

**Affiliations:** ^1^ Department of Research, Mayo Clinic in Arizona, Scottsdale, Arizona, United States of America; ^2^ Biodesign Institute, Arizona State University, Tempe, Arizona, United States of America; ^3^ Department of Pathology, Dunedin School of Medicine, University of Otago, Dunedin, New Zealand; ^4^ Division of General Surgery, Section of Surgical Oncology, Mayo Clinic in Arizona, Phoenix, Arizona, United States of America; ^5^ Section of Biostatistics, Mayo Clinic in Arizona, Scottsdale, Arizona, United States of America; ^6^ Department of Pathology and Laboratory Medicine, Mayo Clinic in Arizona, Scottsdale, Arizona, United States of America; ^7^ Vanderbilt University, Nashville, Tennessee, United States of America; ^8^ Division of Hematology-Oncology, Mayo Clinic in Arizona, Scottsdale, Arizona, United States of America

**Keywords:** 9p24.1 amplicon, flow sorting, triple negative breast cancer, JAK2, PD-L1

## Abstract

We used DNA content flow cytometry followed by oligonucleotide array based comparative genomic hybridization to survey the genomes of 326 tumors, including 41 untreated surgically resected triple negative breast cancers (TNBC). A high level (log_2_ratio ≥1) 9p24 amplicon was found in TNBC (12/41), glioblastomas (2/44), and colon carcinomas (2/68). The shortest region of overlap for the amplicon targets 9p24.1 and includes the loci for *PD-L1*, *PD-L2*, and *JAK2* (PDJ amplicon). In contrast this amplicon was absent in ER+ (0/8) and HER2+ (0/15) breast tumors, and in pancreatic ductal adenocarcinomas (0/150). The PDJ amplicon in TNBCs was correlated with clinical outcomes in group comparisons by two-sample *t*-tests for continuous variables and chi-squared tests for categorical variables. TNBC patients with the PDJ amplicon had a worse outcome with worse disease-free and overall survival. Quantitative RT-PCR confirmed that the PDJ amplicon in TNBC is associated with elevated expression of JAK2 and of the PD-1 ligands. These initial findings demonstrate that the PDJ amplicon is enriched in TNBC, targets signaling pathways that activate the PD-1 mediated immune checkpoint, and identifies patients with a poor prognosis.

## INTRODUCTION

There is an emerging recognition of the role of immune checkpoints in the pathogenesis of solid tumors [1]. Distinct inhibitory pathways serve to regulate T cell activation and function. These include the immune regulatory molecules PD-L1 and PD-L2 that limit the duration and the level of the T-cell response. Increased expression of these ligands of the immune checkpoint receptor PD-1 has been reported in human tumors [2–4]. PD-1 is a CD28 and CTLA-4 homologue that is normally induced on activated T cells, but the chronic antigenic exposure in cancer may lead to high levels of PD-1 and T cell exhaustion [5]. Consequently PD-1 and its ligands are being investigated as candidate biomarkers for response to targeted immune checkpoint blockade in clinical trials of an increasing variety of tumors. Two PD-1 inhibitors, nivolumab and pembrolizumab, have been approved for clinical use in melanoma and are in clinical trials in other solid tumors [6]. In melanoma, the combination of nivolumab plus ipilimumab which targets the PD-1 homologue CTLA-4 was associated with a > 80% decline in tumor burden at 12 weeks in respondents and a 53% overall response rate (ORR) [7]. More recent studies with a larger cohort of patients with untreated metastatic melanoma reported an ORR of 61% for the combination therapy with complete responses in 22% of patients [8]. However clinical studies have been limited by the lack of predictive biomarkers of disease response, the complexity of tumor genomes, and the degeneracy of the receptor/ligand interactions.

The Janus kinase 2 gene (JAK2) is one of four members of the JAK family (which includes JAK1, JAK2, JAK3 and non-receptor protein-tyrosine kinase 2 (TYK2) [9]. JAKs associate with the cytoplasmic portion of a variety of transmembrane cytokine and growth factor receptors important for signal transduction in hematopoietic cells. Receptor binding by extracellular ligand causes receptor multimerization and brings JAK proteins together to allow activation by transphosphorylation. Activated JAK2 has emerged as an important target in myeloproliferative disorders, and increasingly, in solid tumors [10]. Significantly JAK2 has been implicated in interleukin (IL)-6-dependent breast cancer stem cell self-renewal [11], and in both IL-6- and IL-8-dependent growth of triple-negative breast cancers (TNBCs) [12]. Pre-clinical studies have implicated JAK2 signaling as a mechanism of escape from targeted therapies in TNBC and as a promoter for the emergence of more invasive tumor cells [13]. Thus, JAK2 inhibitors are being evaluated in patients with breast and other solid tumors. However a case control study of 223 breast tumors reported an association between increased JAK2 mRNA levels and favorable prognosis [14], and a strong correlation between JAK2 mRNA levels with the presence of tumor-infiltrating lymphocytes (TILs). The protective effect of elevated JAK2 expression was not associated with increased JAK2 protein levels in the tumor epithelial cells. These observations suggest that therapeutic targeting of JAK2 expression may abrogate the potential benefits arising from an antitumor immune response. Thus the clinical significance of selective amplification and overexpression of JAK2 arising from the often highly aberrant genomic landscapes of solid tumors remains to be elucidated.

The PD-L1 and PD-L2 genes localize to 9p24.1 adjacent to JAK2 and there is emerging data that an amplicon targeting this 9p24.1 locus is present in lymphomas and a subset of EBV-positive gastric cancers [15, 16]. Furthermore JAK2 has been shown to up-regulate the transcription of both PD-1 ligands and increase sensitivity to JAK2 inhibitors in a dose dependent manner [16]. We hypothesized that targeted amplification of 9p24.1 in tumor genomes would result in co-amplification of PD-L1, PD-L2, and JAK2, identify a distinct molecular subtype arising in multiple cancers, and provide a candidate biomarker for patients who may benefit from immune checkpoint targeted therapies. In order to address tissue and clonal heterogeneity in clinical samples we used DNA content based flow sorting to identify and purify distinct tumor populations in each tissue of interest [17–19]. There are well-established DNA staining based methods for isolating nuclei of aneuploid, tetraploid, and diploid neoplastic populations from solid tumor samples [20–23]. Individual populations of cells or nuclei can be objectively and quantitatively purified to greater than 95% purity for molecular analyses even in heavily admixed and sub-optimal clinical samples. We have developed and extensively validated flow cytometry based methodologies to study a wide variety of clinical samples including small needle biopsies, post treatment tissues, and formalin fixed paraffin embedded (FFPE) tissues with high definition genome assays such as oligonucleotide based array comparative genomic hybridization (aCGH) and next generation sequencing NGS [18, 24].

To test our hypothesis we surveyed the genomes of 326 clinical samples representing pancreatic ductal adenocarcinomas (PDA) (*n* = 150), glioblastomas (*n* = 44), colorectal carcinomas (*n* = 68), and breast carcinomas (*n* = 64). Each tumor sample in this study was flow sorted prior to copy number analysis with oligonucleotide based aCGH. These data were then used to determine the prevalence of amplification of PD-L1, PD-L2, and JAK2 at chromosome 9p24.1 within each tumor type. The presence of this “PDJ” amplicon was then correlated with expression of JAK2 and the PD-1 ligands, and to clinical outcomes in a subset of patients.

## RESULTS

**Figure 1:**
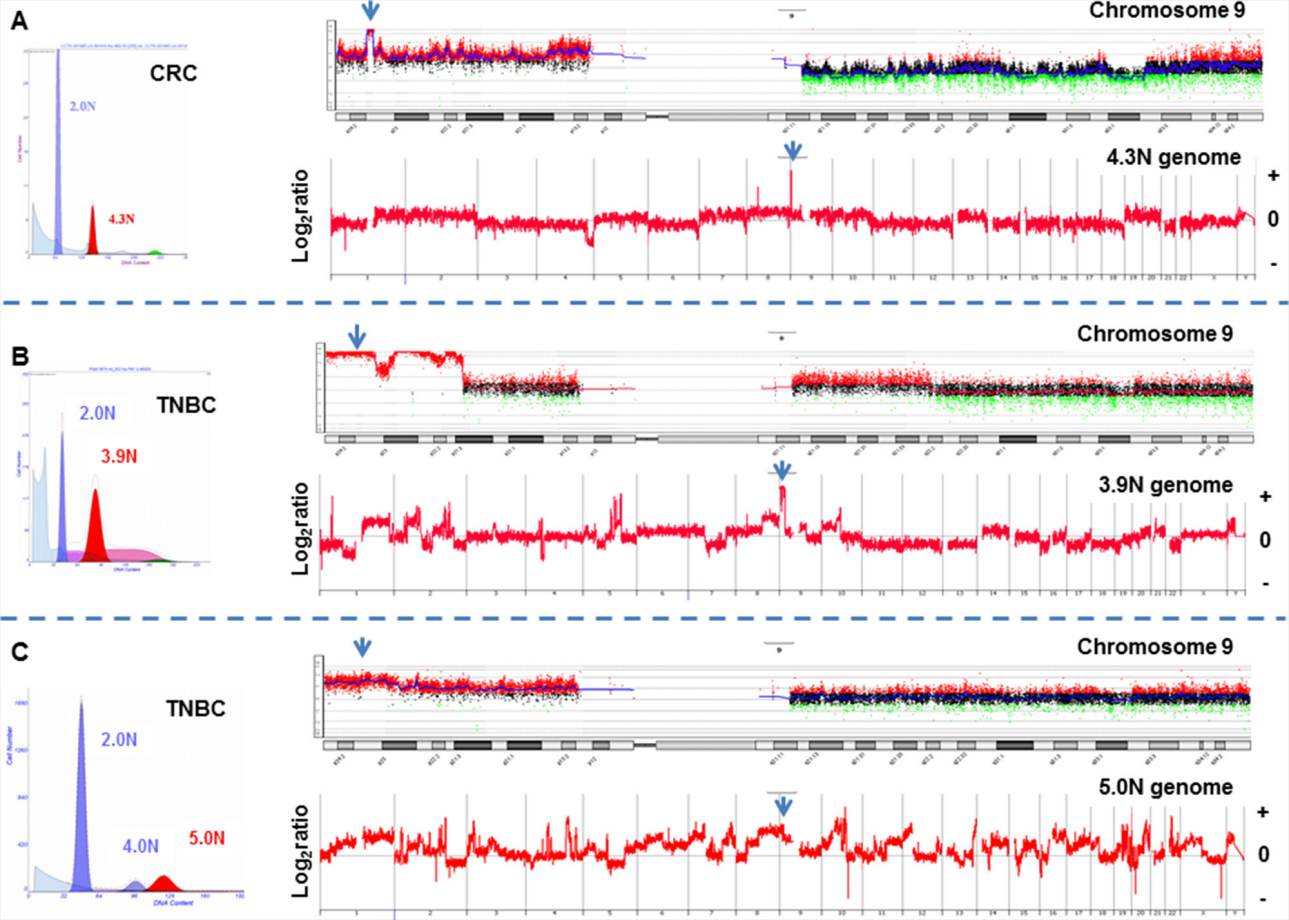
Whole genome and chromosome 9 aCGH plots of flow sorted tumor populations **A.** Colorectal (CRC) and **B–C.** triple negative breast cancers (TNBC) with high level 9p24.1 amplicon. Amplicons were scored according to log_2_ratios >1. Blue arrows denote *JAK2* locus.

**Figure 2:**
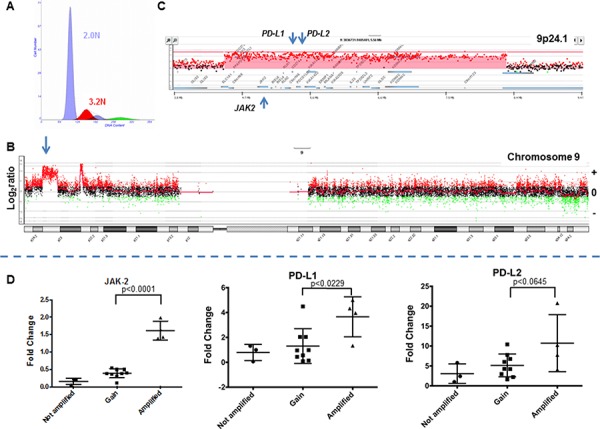
The 9p24 amplicon in a triple negative breast cancer genome **A.** Flow histogram of sorted 3.2N TNBC population from FFPE tissue. **B.** Chromosome 9 CGH plot and detection of 9p24 amplicon. **C.** Gene specific view of amplicon. Red shaded area denotes ADM2 defined copy number aberrant region. **D.** Gene expression of *JAK2*, *PD-L1*, and *PD-L2* in TNBC. Comparisons and correlations between the expression levels of *PD-L1*, *PD-L2*, and *JAK2* genes and copy number status of chromosome 9p24.1 were performed using an unpaired *t* test and variation among and between groups were calculated using an ANOVA test (GraphPad Prism 6).

We detected and sorted an aneuploid and/or a proliferating tumor fraction from each clinical sample in this study. These fractions were gated during sorting providing highly purified and objectively defined tumor populations from each sample for analysis. The tissues included triple negative breast cancer *n* = 41, HER2+ breast cancer *n* = 15, ER+HER2- breast cancer *n* = 8, pancreatic adenocarcinoma *n* = 150 (including 30 liver metastases), colorectal carcinoma *n* = 68, and glioblastoma *n* = 44. These included both fresh frozen and formalin fixed paraffin embedded (FFPE) clinical samples. The tumor cellularity prior to sorting varied extensively from less than 10% to greater than 70% across tissue types. The genomes of each sorted tumor cell population were interrogated with whole genome oligonucleotide based aCGH. Copy number aberrant intervals were identified and their genomic boundaries mapped using a step gram algorithm [25]. Amplicons were then ranked within each sample based on their fold change and their overall prevalence in tumor genomes. A recurring top ranked and high level (log_2_ratio ≥1) amplicon that targeted 9p24.1 was detected in 12/41 TNBCs, 2/68 colon carcinomas, and 2/44 glioblastomas (Figs. 1 and 2). In contrast this amplicon was absent in ER+ (*n* = 8) and HER2+ (*n* = 15) breast tumors, and in pancreatic ductal adenocarcinomas (*n* = 150). The shortest region of overlap (SRO) spanned 777 kb and included the PD-1 ligands PD-L1, PD-L2, and the Janus kinase 2 (JAK2) loci (Fig. S1). The height of this recurring amplicon included mean log_2_ratios >4 consistent with amplification of genomic drivers such as HER2 and MYC described in breast cancer and other solid tumor genomes.

In order to determine the effect of the PDJ amplicon on JAK2, PD-L1, and PD-L2 expression we selected 31 TNBC samples, 16 of which were profiled in our copy number analysis, for qRT-PCR analysis. We used a pooled sample comprised of an unrelated normal breast, and individual TNBC, ER+, and HER2+ tumor tissues to generate a standard curve for assaying JAK2, PD-L1 and PD-L2 expression in our TNBC cohort. Tumors with a high level amplicon (4/16 TNBCs surveyed by qRT-PCR) had significantly higher expression of JAK2 and PD-L1 genes compared to those without the amplicon (Fig. 2). The latter included samples with low level copy number gains (log_2_ratio >0 and <1) at 9p24.1 including increases of whole 9p arm and polysomy of chromosome 9. In addition we identified another TNBC in the subset of 15 tumors without aCGH data with concurrent elevated expression of JAK2 and PD-L1 (Fig. S2). PD-L2 expression was also elevated in the presence of the PDJ amplicon however it did not reach statistical significance (*p* < 0.0645) in this preliminary study. These observations are consistent with studies showing that genomic amplification of 9p24.1 leads to coordinated overexpression of these genes in human tumors [15, 16].

**Figure 3:**
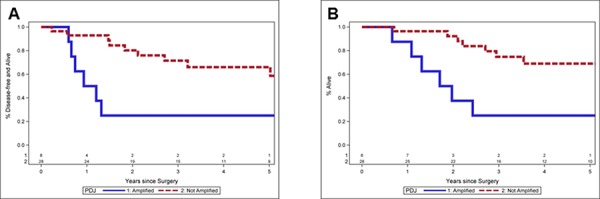
Clinical outcomes for TNBC patients with or without PDJ amplicon **A.** Progression free survival. Lower disease-free survival at 5 years 25.0% vs. 66.0%, *p* = 0.005. **B.** Overall survival. Lower overall survival at 5 years 25.0% vs. 69.0%, *p* = 0.004. Median follow up is 4.7 years (range 0.9–12.0 years).

Clinical data was available on 36 of 41 (88%) of the TNBC patients that were flow sorted then profiled for copy number (Table 1). Patients with the high level PDJ amplicon (*n* = 8) were noted to have larger tumors (mean 3.9 cm vs. 1.9 cm, *p* = 0.04) and a higher incidence of lymph node metastases (75% vs. 26%, *p* = 0.01). Lymphocytic infiltration was noted in 4 of the 36 patients, none of whom had the PDJ amplicon in their tumor genome. Twenty nine of these 36 TNBC patients received chemotherapy after definitive surgical therapy. The disease-free survival rate at 5 years was 25% in the PDJ amplified patients, and 66% in the unamplified patients (*p* = 0.005) (Fig. 3). Overall survival (OS) at 5 years was 25% in the PDJ amplified patients, compared with 69% in the unamplified patients (*p* = 0.004). Thus our preliminary results suggest that the presence of the PDJ amplicon defines a clinically significant subset of high-risk TNBC patients.

**Table 1:** Comparison between patients with and without PDJ amplification

	Not Amplified (*N* = 28)	Amplified (*N* = 8)	Total (*N* = 36)	*p* value
**Age [years]**				0.79^1^
Mean (SD)	53.4 (12.88)	54.8 (8.71)	53.7 (11.98)	
Median	54.0	53.5	54.0	
Range	(29.0–78.0)	(45.0–72.0)	(29.0–78.0)	
**Tumor Size [cm]**				0.04^1^
Mean (SD)	1.9 (0.86)	3.9 (3.88)	2.4 (2.11)	
Median	1.9	2.5	2.0	
Range	(0.5–4.0)	(0.4–11.0)	(0.4–11.0)	
**Grade**				0.63^2^
1	1 (3.6%)	0 (0%)	1 (2.8%)	
2	7 (25%)	1 (12.5%)	8 (22.2%)	
3	20 (71.4%)	7 (87.5%)	27 (75%)	
**Lymphocytic Infiltrates**				0.21^2^
Missing	10 (.%)	2 (.%)	12	
No	14 (77.8%)	6 (100%)	20 (83.3%)	
Yes	4 (22.2%)	0 (0%)	4 (16.7%)	
**Lymph Nodes**				0.01^2^
Missing	1 (%)	0 (.%)	1	
Negative	20 (74.1%)	2 (25%)	22 (62.9%)	
Positive	7 (25.9%)	6 (75%)	13 (37.1%)	
**Cancer Stage**				0.04^2^
Missing	1 (.%)	0 (.%)	1	
Stage I	11 (40.7%)	1 (12.5%)	12 (34.3%)	
Stage II	13 (48.1%)	3 (37.5%)	16 (45.7%)	
Stage III	3 (11.1%)	4 (50%)	7 (20%)	
**Surgical Treatment**				0.70^2^
Missing	9 (.%)	2 (.%)	11	
Breast Conservation Therapy (BCT)	8 (42.1%)	2 (33.3%)	10 (40%)	
Mastectomy	11 (57.9%)	4 (66.7%)	15 (60%)	
**Chemotherapy**				0.10^2^
Neoadjuvant	1 (3.6%)	2 (25%)	3 (8.3%)	
No	4 (14.3%)	0 (0%)	4 (11.1%)	
Yes	23 (82.1%)	6 (75%)	29 (80.6%)	
**Radiation [Mastectomy only]**				0.88^2^
Missing	17 (.%)	4 (.%)	21	
No	5 (45.5%)	2 (50%)	7 (46.7%)	
Yes	6 (54.5%)	2 (50%)	8 (53.3%)	

1 Two-Sample *T*-Test

2 Chi-Square

## DISCUSSION

Studies in gastric cancer and in lymphomas have described a recurring 9p24.1 amplicon that includes PD-L1, PD-L2, and JAK2 [15, 16]. The presence of the amplicon was associated in a subset of each tumor type with distinct pathological and clinical features. Functional studies revealed that JAK2 is a transcriptional activator of both PD-1 ligands [16] and that sensitivity to JAK2 inhibition occurs in a dose-dependent manner [26]. Thus genomic amplification of 9p24.1 may provide a selective tumor cell dependent increase in both JAK2 and immune checkpoint signaling. In addition, copy number gains on chromosome 9 that include 9p24.1 and elevated expression of PD-L1 within tumor cells have recently been associated with substantial therapeutic activity in patients with advanced or refractory Hodgkin's lymphoma treated with the PD-1 inhibitor nivolumab [27]. Notably, two early phase I trials of PD-1/PD-L1 blockade (pembrolizumab or MPDL3280A) in TNBC have demonstrated overall response rates of 15–20% [28, 29].

PD-L1 expression across multiple solid tumors varies significantly by tumor type and appears common in both tumor-infiltrating immune cells and in tumor cells [4, 30]. Elevated PD-L1 expression from tumor infiltrating immune cells had a stronger association with clinical response than that observed with expression from the tumor cells [4]. Significantly elevated expression of JAK2 in the non-epithelial compartment of breast tumors has been associated with a better prognosis and decreased risk of recurrence [14]. Thus an important clinical question is the role of a tumor cell driven elevation in JAK2 and PD-1 ligands versus increases arising from immune cells in solid tissue cancers [6, 27].

Previous *in vitro* studies of a 9p amplicon in a small panel of established breast cancer cell lines excluded JAK2, PD-L1, and PD-L2 loci from the SRO reported [31]. However JAK2 copy number levels assessed in bulk TNBC biopsies by targeted resequencing of a panel of 196 cancer associated genes have been reported to be elevated in residual disease post neoadjuvant chemotherapy. The JAK2 amplification was associated with poor regression free survival (RFS) and predicted poor OS in a subset (~10%) of TNBCs [32]. The prevalence of JAK2 amplification was noted to be higher in these post treatment TNBCs than reported for treatment naïve TNBCs in TCGA. The latter are based on single nucleotide polymorphism (SNP) array data of bulk tumor samples. Consequently it remained to be resolved whether JAK2 amplifications and the potential co-amplifications of PD-L1 and PD-L2 are present in untreated TNBCs, and whether genomic amplification at 9p24.1 may provide a biomarker for clinical outcome. Furthermore recent correlative studies with FISH based markers in lymphoma have proposed that even tumors with low level 9p gains and chromosome 9 polysomy may benefit from PD-1 targeted therapies [27].

Our preliminary results with highly purified flow sorted clinical samples and an array platform designed for whole genome copy number measures suggest that the PDJ amplicon has a SRO that targets JAK2 and the PD-1 ligands, is enriched in a subset of TNBCs with poor outcomes prior to therapy, and is distinct from background genomic aberrations affecting chromosome 9. Given the coordinated overexpression of PD-L1, PD-L2, and JAK2, antagonists targeting PD-1 signaling and JAK2 inhibitors should be evaluated in the context of PDJ amplification. Thus we propose that the PDJ amplicon provides a candidate biomarker for identifying high-risk patients and for advancing emerging immunotherapies in TNBC. The absence of this amplicon in pancreatic adenocarcinoma samples and its relatively low frequencies in colorectal cancers and glioblastomas are consistent with relatively low response rates reported in these and other solid tumors when compared to TNBC [33, 34]. However the presence and role of the PDJ amplicon in a smaller subset (2–5%) of colorectal cancers, including primary and matching lymph node biopsies (Fig. S3), and glioblastomas warrants further investigation.

**Figure 4:**
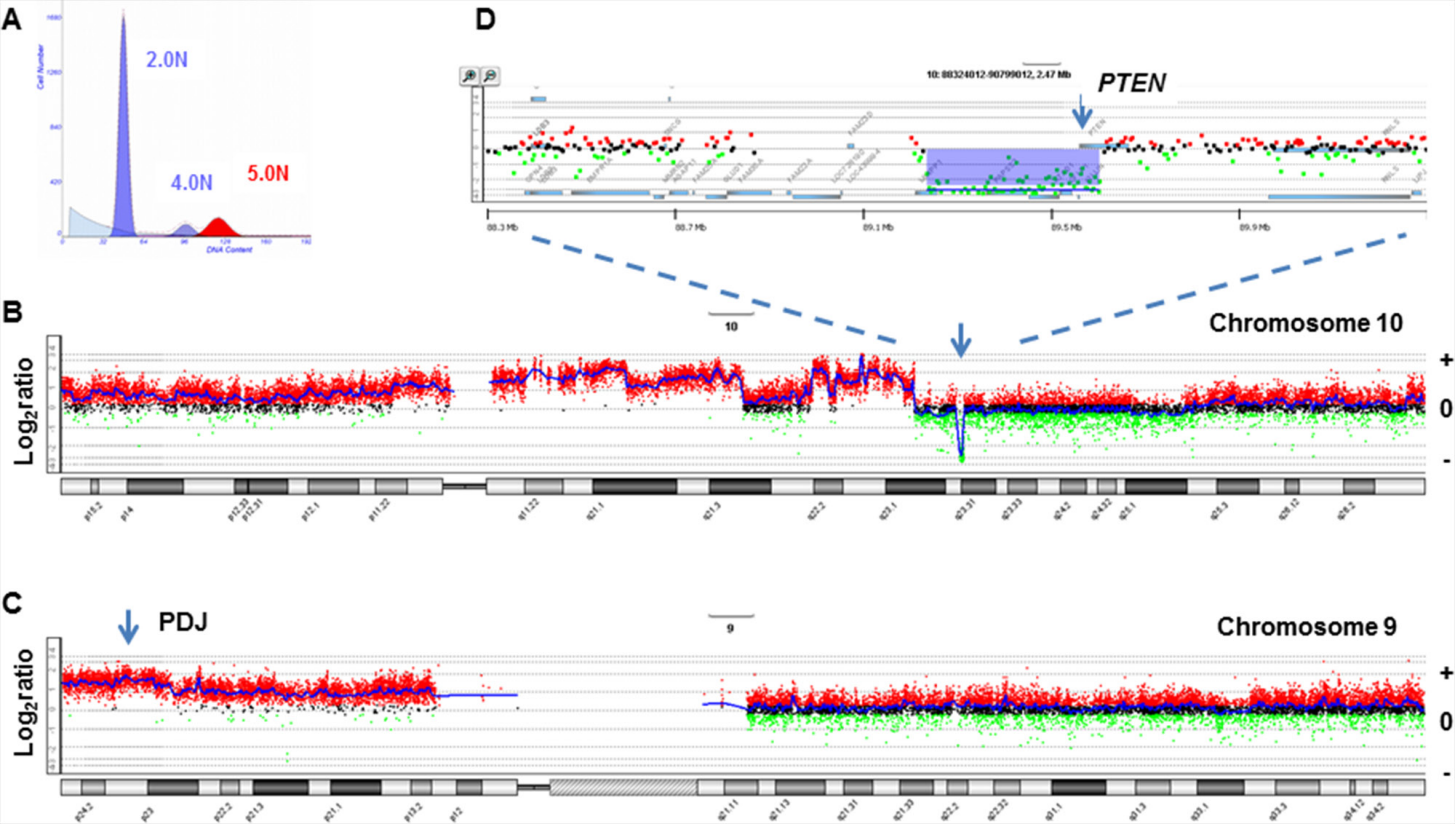
PTEN homozygous deletion in PDJ^+^ triple negative breast cancer genome **A.** Flow histogram of sorted 5.0N TNBC population from FFPE tissue. **B–C.** Chromosome 10 and chromosome 9 CGH plots. **D.** Gene specific view of PTEN homozygous deletion. Blue shaded area denotes ADM2 defined copy number aberrant region.

Given the highly aberrant genomic landscapes of TNBCs and other solid tumors, alterations that disrupt immune regulatory signaling pathways upstream or downstream of PDJ amplicon may provide additional biomarkers for this emerging class of therapies [35]. Studies in cell line models suggest that deletions in PTEN and disruption of AKT signaling can increase PD-L1 levels [36]. Our use of flow sorted clinical samples provides a robust objective method to detect somatic lesions targeting clinically actionable pathways [18, 24]. For example we detected focal deletions of PTEN in the tumor genomes of 5/12 PDJ positive TNBCs (Fig. 4 and Fig. S4). Future studies will incorporate flow sorted tissue samples and both copy number and next generation sequencing analyses to provide a more comprehensive and unbiased profile of the genomic landscapes of tumors with the PDJ amplicon. Of significant interest will be biopsies from patients enrolled in clinical trials with agents that target the PD-1 immune checkpoint. The ability to discriminate mutations and genomic lesions including the PDJ amplicon arising in the epithelial component of solid tumors and to comprehensively interrogate genomes in the pre and post adjuvant setting, regardless of tumor cell content, will be essential to understand clinical responses and to advance effective immunotherapies. These data will also be used to develop robust clinically relevant assays (e.g. targeted resequencing, FISH, IHC) to efficiently identify those patients with the PDJ amplicon. We propose that the PDJ amplicon in TNBC and other solid tumors represents a novel candidate biomarker with broad application for cancer research and for advancing personalized therapies for cancer patients.

## MATERIALS AND METHODS

### Clinical samples

TNBC, ER+, and HER2+ samples were obtained under approval from the Mayo Clinic Institutional Review Board prior to undertaking this study (IRB protocol 08-006579). Tumor specimens were obtained from formalin fixed paraffin embedded (FFPE) archived breast cancer samples obtained at the time of definitive surgical resection. All breast cancers underwent central pathologic review, and were evaluated by IHC for estrogen receptor (ER), progesterone receptor (PR), and by IHC +/− FISH for Her2/neu under CLIA/CAP guidelines. TNBC samples were also obtained from The Inflammatory Breast Cancer Research Foundation (IBCRF) Biobank. Samples were collected following informed consent by the IBCRF under approval of their IRB and their Medical Advisory Board. Additional flow sorted TNBC data was from a previous study of breast cancer genomes that was performed with informed consent and ethics committee approvals [37]. PDA samples were from previous studies obtained under a WIRB protocol (20040832) for an NIH funded biospecimen repository (NCI P01 Grant CA109552), Stand up To Cancer clinical trials 2026001 and 2026003, and with approved consent of the Ethics Committee of Basel (252/08, 302/09). The glioblastoma samples were collected for another previous study with informed consent from patients at the Neurosurgery Department of the Centre Hospitalier in Luxembourg (CHL) [38]. Samples were approved for study by the National Ethics Committee for Research (CNER) of Luxembourg. In all cases written consent was obtained. De-identified colon samples did not qualify as human subjects according to guidelines administered by the Translational Genomics Research Institute (TGen) Office of Research Compliance and were collected under an institutional blanket exemption as part of different research initiatives and from the Cooperative Human Tissue Network (CHTN).

### FFPE sample preparation

Prior to sorting, excess paraffin was removed with a scalpel from either side of 40–60 um scrolls then processed according to our published methods [24]. Briefly each sectioned piece was collected into individual microcentrifuge tubes then washed three times with 1 ml Xylene for 5 minutes to remove remaining paraffin. Each sample was rehydrated in sequential ethanol washes (100% 5 minutes × 2, then 95%, 70%, 50% and 30% ethanol) and washed 2 times in 1 ml 1 mM EDTA pH 8.0. A 1 ml aliquot of 1 mM EDTA pH 8.0 was added to the samples and incubated at 95°C for 80 minutes to facilitate the removal of protein cross-links present in FFPE tissue. Samples were then cooled to room temperature for ≥5 minutes, followed by addition of 300 ul PBS pH 7.4 and gentle centrifugation for 2 minutes at 3.6 × g. The supernatant was carefully removed and the pellet washed three times with 1 ml PBS pH 7.4/0.5 mM CaCl_2_ to remove EDTA. Each sample was digested overnight (6–17 hours) in 1 ml of a freshly prepared enzymatic cocktail containing 50 units/ml of collagenase type 3, 80 units/ml of purified collagenase, and 100 units/ml of hyaluronidase in PBS pH 7.4/0.5 mM CaCl_2_ buffer. Following overnight digestion 500 ul NST buffer (146 mM NaCl, 10 mM Tris-HCl, pH 7.5, 1 mM CaCl_2_, 0.5 mM MgSO_4_, 21 mM MgCl_2_, 0.05% bovine serum albumin, 0.2% Nonidet P40 (Sigma)) with 4, 6-diamindino-2-phenylindole (DAPI; 10 μg/ml) was added to each sample to facilitate pelleting. Samples were centrifuged for 5 minutes at 3000 × g, after which pellets were resuspended in 750 ul of NST/10% fetal bovine serum and then passed through a 25 G needle 10–20 times. We used a single 50 μm scroll from each FFPE tissue block to obtain sufficient numbers of intact nuclei for subsequent sorting and molecular assays.

### Flow cytometry

Biopsies were minced in the presence of NST buffer and DAPI according to published protocols [23, 39, 40]. Prior to sorting each sample was filtered through a 35 um mesh and collected into a 5 ml Polypropylene round bottom tube. The mesh was rinsed with an additional 750 ul of NST/10% fetal bovine serum and placed on ice while processing remaining samples. The total volume in the tube for each sample was approximately 1.5 ml. An equal volume of 20 ug/ml DAPI was added to each tube to achieve a final concentration of 10 ug/ml DAPI for flow sorting with a BD Influx cytometer with ultraviolet excitation (Becton-Dickinson, San Jose, CA). The optimal settings for sorting FFPE samples with the Influx sorter were as follows: Drop formation was achieved with piezzo amplitude of 6–10 volts and a drop frequency of 30 khertz. The sort mode was set to purity yield with a drop delay of 31.5–32. Sheath fluid pressure was typically 17–18 psi with a 100 um nozzle. For single parameter DNA content assays DAPI emission was collected at >450 nm. DNA content and cell cycle were then analyzed using the software program MultiCycle (Phoenix Flow Systems, San Diego, CA).

### DNA extraction

DNA from sorted nuclei was extracted using an amended protocol from QIAamp^®^ DNA Micro Kit from Qiagen (Valencia, CA). Briefly each sorted sample was resuspended in 180 ul buffer ATL and 20 ul proteinase K (20 mg/ml) then incubated for 3 hours at 56°C for complete lysis. Samples were bound and washed according to QIAamp^®^ DNA Micro Kit instructions, eluted into 50 ul of H_2_0, then precipitated overnight with 5 ul 3 M sodium acetate and 180 ul 100% EtOH. Each sample was then centrifuged for 30 minutes at 20, 000 × g, washed in 1 ml of 70% EtOH for 30 minutes at 20, 000 × g. The samples were carefully decanted and the DNA pellet was dried by speed vacuum then resuspended in a small volume (e.g. 10–50 ul) of H_2_0 for final concentrations suitable for accurate quantitation.

### aCGH analysis

DNAs were treated with DNAse 1 prior to Klenow-based labeling. High molecular weight reference templates were digested for 30 minutes while the smaller fragmented FFPE-derived DNA samples were digested for only 1 minute. In each case 1 ul of 10× DNase 1 reaction buffer and 2 ul of DNase 1 dilution buffer were added to 7 ul of DNA sample and incubated at room temperature then transferred to 70°C for 30 minutes to deactivate DNase 1. Sample and reference templates were then labeled with Cy-5 dUTP and Cy-3 dUTP respectively using a BioPrime labeling kit (Invitrogen, Carlsbad, CA) according to our published protocols [18]. All labeling reactions were assessed using a Nanodrop assay (Nanodrop, Wilmington, DE) prior to mixing and hybridization to CGH arrays (Agilent Technologies, Santa Clara, CA) for 40 hours in a rotating 65°C oven. All microarray slides were scanned using an Agilent 2565C DNA scanner and the images were analyzed with Agilent Feature Extraction version 10.7 using default settings. The aCGH data was assessed with a series of QC metrics then analyzed using an aberration detection algorithm (ADM2) [25]. The latter identifies all aberrant intervals in a given sample with consistently high or low log ratios based on the statistical score derived from the average normalized log ratios of all probes in the genomic interval multiplied by the square root of the number of these probes. This score represents the deviation of the average of the normalized log ratios from its expected value of zero and is proportional to the height h (absolute average log ratio) of the genomic interval, and to the square root of the number of probes in the interval. All aCGH data in this paper have been deposited at the National Center for Biotechnology Information Gene Expression Omnibus (accession number in progress).

### Expression analysis

Total RNA was extracted from one whole-tissue 50 μm thick section using RNeasy FFPE RNA Isolation Kit (Qiagen). RNA quantification was performed using Qubit 2.0 fluorometer (Life Technologies) and Qubit RNA HS assay kit (molecular probes). Reverse transcription was carried out using SuperScript^®^ VILO™ cDNA Synthesis Kit (Life Technologies) and 200 ng of RNA per reaction, with triplicate reactions performed for each sample [41]. Each of the 31 samples produced sufficient RNA yield and quality. Quantitative Real-Time PCR was performed for *CD274*, *PDCD1LG2*, and *JAK2* (Hs01125301_m1, Hs01057777_m1 Hs00234567_m1, respectively; Life Technologies) with TaqMan^®^ chemistry on the ABI Prism 7900HT (Applied Biosystems), using the standard curve method. Furthermore, two reference genes were used *TFRC* and *MRPL19* (Hs00174609_m1, Hs00608519_m1, respectively; Life Technologies), previously selected as effectively normalizing for degradation of the FFPE-RNA. Target gene expression levels were normalized to the geometric mean of the two reference genes and normalized to a pool of RNAs prepared from a normal and from 3 FFPE breast tumors (TNBC, ER+, and HER2+). All statistical comparisons and correlations between the expression levels of *PD-L1*, *PD-L2*, and *JAK2* genes and copy number status of chromosome 9p24.1 were performed using an unpaired *t* test and variation among and between groups were calculated using an ANOVA test (GraphPad Prism 6). The *p*-values < 0.05 were considered significant.

### Statistical analysis

Group comparisons used two-sample *t*-tests for continuous variables and chi-squared tests for categorical variables. Disease-free survival time was defined as the time from primary surgery to first local, regional, or distant recurrence or death regardless of cause. Overall survival analysis included all deaths as events regardless of cause. Disease-free and overall survival rates at 5 years were estimated using the method of Kaplan and Meier. Disease-free survival and overall survival were compared between groups using a log-rank test. Median follow-up was 4.7 years (range 0.9–12.0 years). *P*-values < 0.05 were considered statistical significant throughout.

## SUPPLEMENTARY FIGURES


