# Supplementary material for: Genomic amplification of 9p24.1 targeting JAK2, PD-L1, and PD-L2 is enriched in high-risk triple negative breast cancer
Source: Oncotarget. 2015 Jul 3;6(28):26483–93. doi: 10.18632/oncotarget.4494 (PMC4694916; doi:10.18632/oncotarget.4494)
Supplement: Supplementary file 1 [file oncotarget-06-26483-s001.pdf]

SUPPLEMENTARY FIGURES

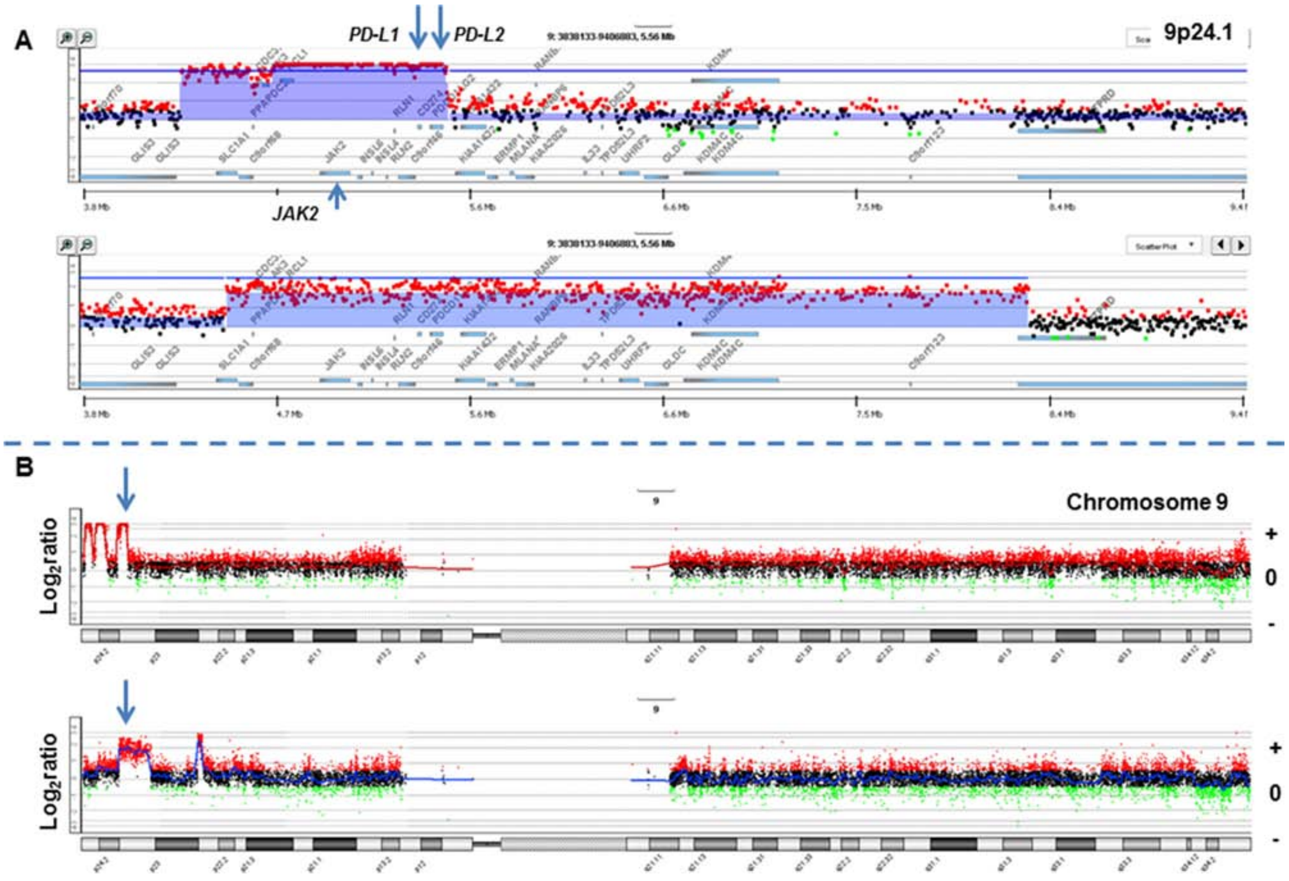

Supplementary Figure S1: Mapping the shortest region of overlap (SRO) of the PDJ amplicon. Chromosome 9 CGH plots of high level and focal 9p24.1 amplicon in colorectal (top panel A, B) and breast (bottom panel A, B) cancer genomes. Shaded areas denote ADM2 defined copy number aberrant intervals.

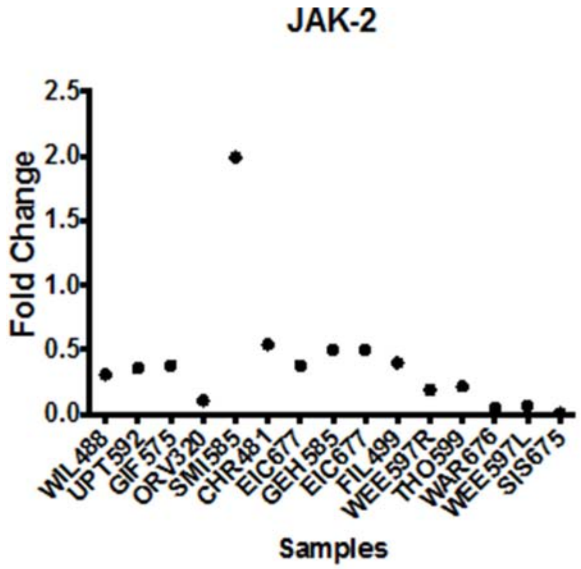

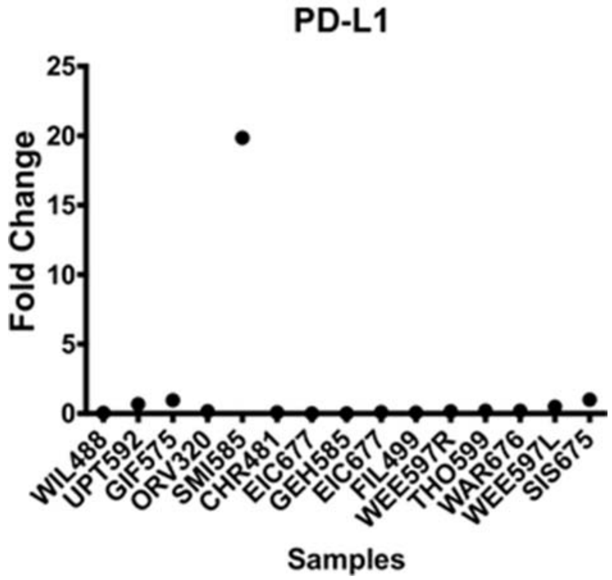

Supplementary Figure S2: Gene expression analysis of *JAK2*, and *PD-L1* in 15 TNBCs without aCGH data. Target gene expression levels were normalized to the geometric mean of the two reference genes and normalized to a pool of RNAs prepared from a normal and from 3 FFPE breast tumors (TNBC, ER+, and HER2+). TNBC SMI585 had co-occurring elevated expression of *JAK2* and PD-L1.

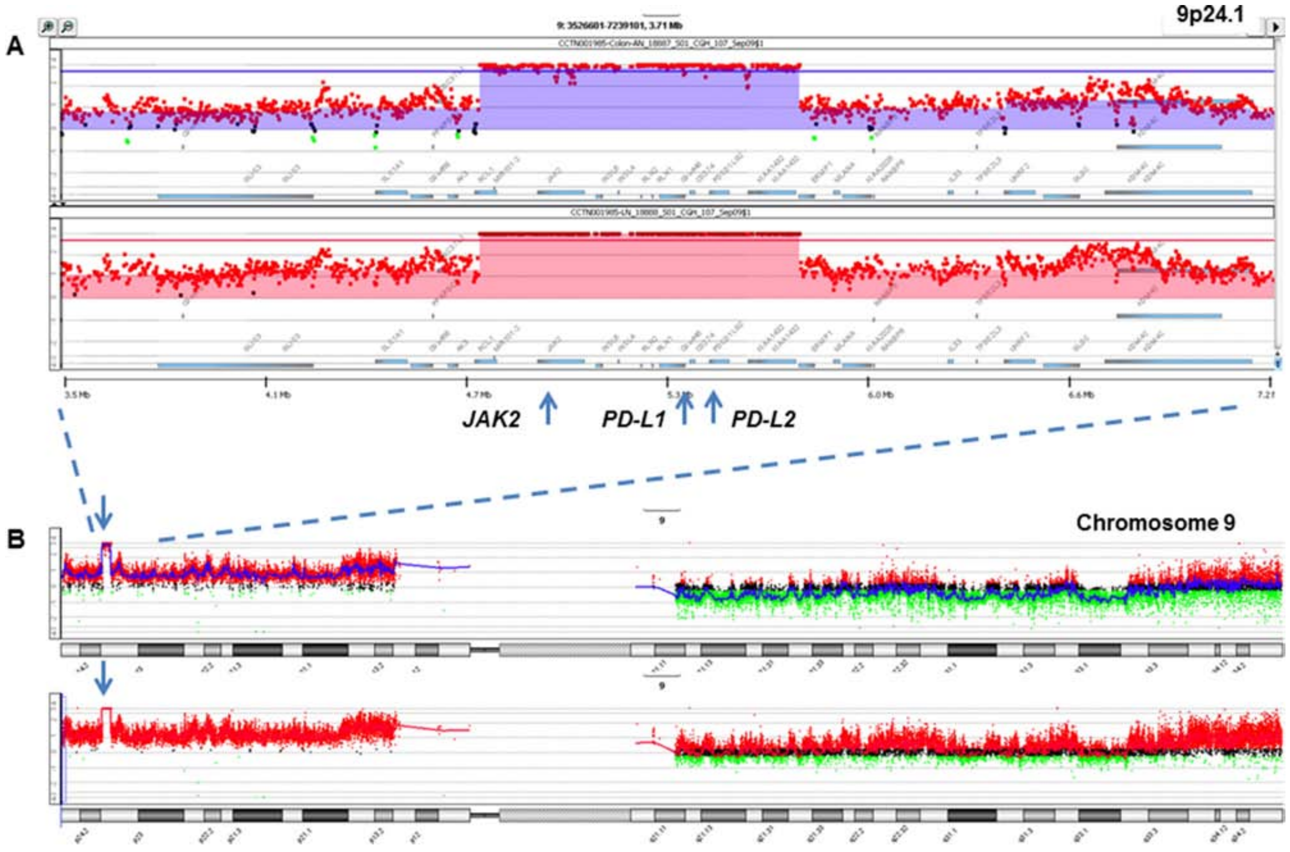

Supplementary Figure S3: PDJ amplicon present in primary colorectal carcinoma and matching lymph node biopsies. A. Gene specific view of shared 9p24.1 amplicon in primary (top) and lymph node (bottom). **B.** Chromosome 9 CGH plots of primary (top) and lymph node (bottom). Blue and red shaded area denotes ADM2 defined copy number aberrant region.

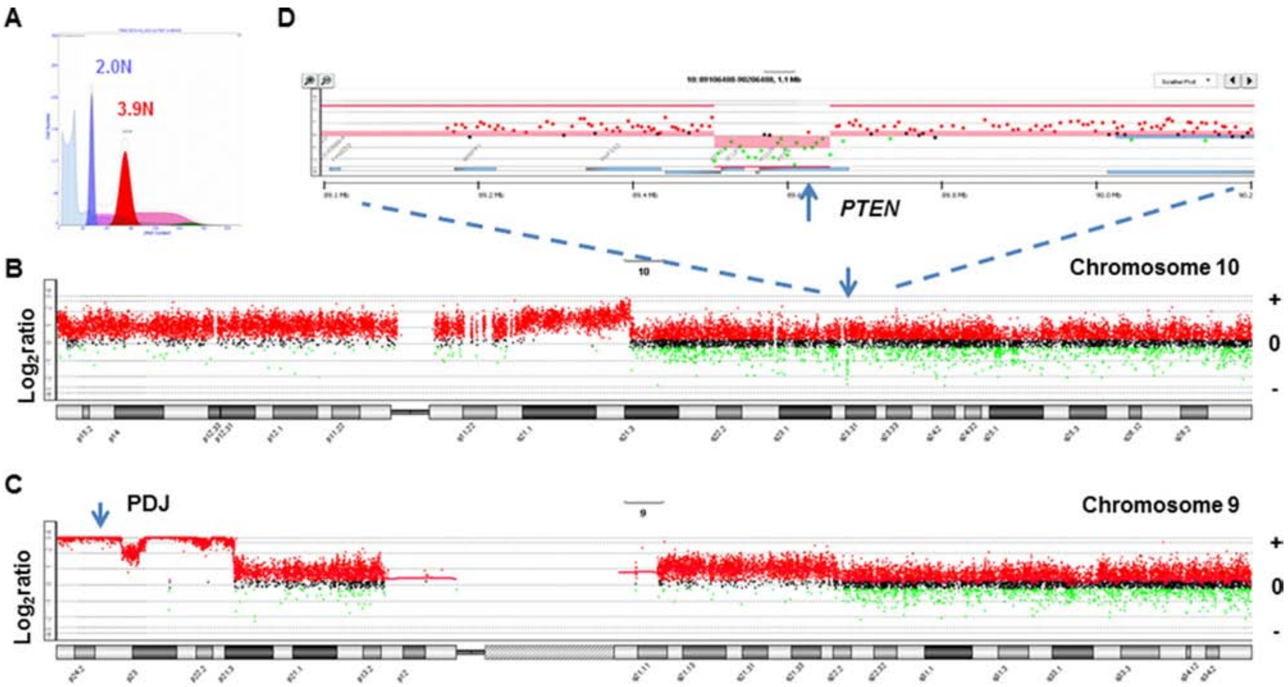

Supplementary Figure S4: PTEN deletion in a PDJ⁺ triple negative breast cancer genome. A. Flow histogram of sorted 3.9N TNBC population from FFPE tissue. **B–C.** Chromosome 10 and chromosome 9 CGH plots. **D.** Gene specific view of PTEN deletion. Blue shaded area denotes ADM2 defined copy number aberrant region.
